# Transformational Leadership Styles, Adolescent Burnout, and the Mediating Role of Basic Psychological Needs: A Cross-Sectional Study on Family and Sport Contexts

**DOI:** 10.3390/sports14020048

**Published:** 2026-02-02

**Authors:** Nerea Torres-Moya, Lucía Arias-Casasús, Ignacio Celsi, Inés Tomás, Isabel Castillo, Octavio Alvarez

**Affiliations:** 1 Department of Social Psychology, University of Valencia, 46010 Valencia, Spain; tomone@alumni.uv.es (N.T.-M.); luaca4@alumni.uv.es (L.A.-C.); octavio.alvarez@uv.es (O.A.); 2 Advanced Research Group in Psychology of Physical Activity, Exercise, and Sport (GIAPAFED), University of Valencia, 46010 Valencia, Spain; ignaciocelsi@conicet.gov.ar (I.C.); ines.tomas@uv.es (I.T.); 3 National Scientific and Technical Research Council (CONICET), Buenos Aires C1425FQB, Argentina; 4 Research Institute, Faculty of Psychology, University of Buenos Aires, Buenos Aires C1052AAA, Argentina; 5 Department of Methodology of the Behavioral Sciences, University of Valencia, 46010 Valencia, Spain

**Keywords:** transformational coaching, transformational parenting, basic psychological needs, burnout, adolescents, basketball players

## Abstract

The present study examines the relationships between transformational leadership styles (i.e., transformational coaching and transformational parenting), basic psychological needs (BPN) satisfaction and thwarting, and burnout within sports and family settings among a sample of adolescents. Participants were 540 basketball players (184 girls) between 11 and 18 years old, representing five clubs in the province of Valencia, Spain. A non-experimental cross-sectional study was conducted, and structural equation modeling was used to examine the relationships among the study variables. Both coaches’ and mothers’ transformational leadership styles were positively associated with BPN satisfaction and negatively associated with BPN thwarting. Fathers’ transformational parenting was negatively associated with BPN thwarting. BPN satisfaction and thwarting emerge as an indirect mediator between mothers’ transformational parenting and burnout. A direct association of transformational coaching with players’ burnout was supported, as well as being indirectly associated through BPN thwarting. This study suggests that BPN satisfaction and thwarting are the main mechanisms for understanding the development of player burnout. Coaches, within sports settings, and mothers in the family setting, emerge as fundamental figures for understanding the mechanisms of the relationships between transformational coaching and parenting with regard to player burnout.

## 1. Introduction

According to the youth sports literature, socialization contexts, particularly those of a sport and family nature, play a decisive role in shaping adolescent sporting experiences [1,2]. During this developmental stage, marked by an increase in sports participation, the role of coaches and parents is of particular importance, as they act as primary leaders who can either facilitate or hinder the development of positive youth characteristics. Consequently, sports practice can result in optimal developmental experiences (e.g., engagement, enjoyment, and sense of purpose) or, conversely, in negative experiences (e.g., burnout, and intention to drop out), depending on the support and guidance behaviors provided by these leaders [3,4].

Transformational leadership theory [5] outlines leaders by their ability to align individual efforts and interests with a shared collective vision. In sports, this approach aims to empower athletes to become proactive agents of their own sporting and personal development, helping them to cultivate a sense of purpose and confidence that extends beyond immediate goals and outcomes [6]. Within this context, transformational coaches and parents act as positive role models (idealized influence), offering challenges and opportunities for personal growth through team involvement (inspirational motivation). These practices have been shown to enhance athletes’ confidence, stimulate problem-solving and creative participation (intellectual stimulation), and respond to the unique needs and aspirations of each athlete (individualized consideration).

Transformational coaches play a key role in the growth and development of team members, contributing to both collective and individual benefits [6,7]. This coaching style has been associated with a range of optimal individual experiences, including intrinsic motivation, enjoyment, perceived competence, positive affect, happiness, life satisfaction, and subjective well-being [8,9,10,11,12,13] among athletes. For example, Liu and colleagues [10] found, in a sample of adolescent football, basketball, and volleyball players, direct associations between a transformational coaching style and players’ happiness as an indicator of well-being, and indirect associations throughout players’ BPN satisfaction. At the team level, transformational coaching has been associated with greater feelings of cohesion, higher collective efficacy [11], and stronger social identity [14,15]. Conversely, this leadership style has been found to be negatively related to indicators of ill-being, such as negative affect and burnout [13,16].

Transformational parenting behaviors were initially associated with positive youth development experiences, such as healthy eating, self-regulation of physical activity, and life satisfaction [17,18], underscoring these figures as role models for healthy behavior during adolescence. De la Torre Cruz et al. [19] further expanded these findings by identifying a relationship between adolescents’ perception of transformational parenting and their physical self-concept. Additional evidence indicates that this parenting style is positively associated with subjective happiness and psychological well-being and negatively related to distress and burnout in adolescent athletes [20], showing differences according to gender and parental figure (i.e., mother or father). Moreover, this parenting style has been shown to act as a protective factor against burnout in youth athletes [21]. Thus, Alvarez and colleagues [21] found that a mother’s transformational style has a more significant relationship than a father’s with the levels of their children’s burnout, accounting for the mediation of autonomy support between transformational parenting and burnout. Similarly, Celsi et al. [14] found the same differences when examining transformational parenting and the social identity of their adolescent children. These authors attributed the differences among fathers and mothers to the Mediterranean cultural contexts, arguing that in Spanish-speaking cultures, mothers play an active role in providing emotional support for their children’s activities [22].

The influence of social agents on athletes can be examined through the lens of Basic Psychological Needs Theory (BPNT), a sub-theory of the self-determination framework (SDT) [23,24]. This theory posits that there are three basic psychological needs, considered innate and universal, that must be satisfied for individuals to experience optimal well-being. These needs are as follows: (1) autonomy, defined as the need to experience volition, self-direction, and choice; (2) competence, defined as the need to feel a sense of efficacy in interactions within the social context; and (3) relatedness, defined as the need to feel valued and connected with significant others. In contrast, SDT proposes that when needs are thwarted, individuals are more likely to experience psychological distress and dysfunction. Although the concepts of satisfaction and frustration of basic psychological needs (BPN) are antithetical, they are not opposite ends of a continuum. In other words, low satisfaction does not imply frustration, nor does frustration imply low satisfaction. Rather, it is the context, through autonomy support or control, which will promote satisfaction or frustration, respectively [24]. Thus, and also according to SDT, the satisfaction or thwarting of BPN is not just determined by individuals’ competencies. Instead, it is primarily influenced by the social context [23,25].

Transformational leadership and autonomy-supportive behaviors have been shown to share certain similarities [12]. Based on the previous proposal of Sheldon and colleagues [26], who suggested the importance of a leader’s vision and a role model to promote BPN satisfaction, Stenling and Tafvelin [12] highlighted the similarities between the two concepts. These parallels can be attributed to the fact that both theoretical frameworks are established on the notion of human beings from the standpoint of positive psychology, with the former focusing on leadership and the latter on motivation. Thus, leadership conceptualizes influence processes as an inherent feature of the concept itself. Conversely, interpersonal styles constitute a contextual component of the motivational processes elucidated by SDT. Motivational processes account for how contexts are converted through them into individual behavior, while leadership focuses on how the process of influence (leadership) is produced from the context [27]. Consequently, transformational behaviors are theoretically distinct from autonomy-supportive ones. While the same behavior (e.g., seeking an athlete’s perspective) can be labeled simultaneously as intellectual stimulation (leadership) and autonomy-supporting behavior (motivation), influence processes must be studied from the specific variable that considers these processes. Previous literature has shown a positive association between transformational and autonomy-supporting behaviors, as evidenced in the literature on coaches [28,29] and parents [21]. This underscores the significance of considering athletes’ perspectives, initiative, and needs in promoting positive youth development. Furthermore, previous studies support the proposal that BPN function as robust mediators between contextual variables (e.g., leadership) and well-/ill-being [12,30].

Moreover, the extent to which BPN are fulfilled largely depends on whether coaches and parents encourage autonomy or impose controlling conditions that undermine it [24]. In this regard, within the context of adolescent sport, socializing agents play a pivotal role through the provision of autonomy support [25]. In terms of leadership, transformational behaviors encourage autonomy support [21]. When athletes perceive that these agents foster their involvement and intrinsic interest in sport practice, their BPN are more likely to be satisfied [31,32]. In contrast, when athletes perceive their environment to be adopting a controlling style through punishment and/or intimidation, their BPN are more likely to be thwarted [33,34].

Burnout is a multidimensional psychological syndrome that, in the context of sport, is characterized by physical and emotional exhaustion resulting from the psychosocial and physical demands of training and competition. It also involves a reduced sense of accomplishment, reflected in feelings of ineffectiveness and a negative self-evaluation regarding performance and ability. In addition, burnout is associated with sport devaluation, which refers to a negative and detached attitude toward sport, manifested as little or no concern for the activity, one’s performance, and the quality of that performance. This dimension refers to a lack of desire, decreased interest, and neglect of sport [35,36].

Burnout has been shown to have a negative impact on multiple dimensions of well-being and athletic performance. These include symptoms of depression, anxiety, and chronic stress [37]; fatigue and moodiness [38]; lack of control, frustration, and dysfunctional behaviors [39]; tension, sleep disorders, and decreased positive emotions [40]; and decreased performance and dropping out of sports [41]. These consequences show that burnout exerts a detrimental effect not only on the athlete’s immediate performance but also on their psychological balance, personal development, and long-term career continuity.

In this context, preventing burnout is essential to protect athletes’ mental, physical, and motivational health, as well as to ensure sustainable sports practice. Early identification of signs of exhaustion can help prevent chronic ill-being and sport devaluation [36,42]. Effective prevention involves appropriate management of training loads and rest periods, along with the creation of motivational environments that foster autonomy, competence, and social support, factors recognized as protective against psychological exhaustion [40]. Furthermore, a positive sporting climate and a coach–athlete relationship grounded in trust and open communication are associated with greater well-being and resilience [41]. A comprehensive approach to burnout prevention not only reduces the risk of injury or dropout but also promotes overall health, stable performance, and a long-lasting and fulfilling athletic career [39,41].

As previously mentioned, transformational leadership has been identified as a key factor that is positively associated with positive psychological outcomes in athletes and negatively associated with burnout, primarily through the mediation of the satisfaction of BPN. For instance, Stenling and Tafvelin [12] found that the positive association of transformational leadership with athlete well-being was mediated by satisfaction of BPN, suggesting that the beneficial outcomes associated with transformational leadership, at least in part, stem from leaders’ capacity to fulfill their followers’ needs for autonomy, competence, and relatedness. In a similar vein, Yildirim and Koruç’s [30] study found that transformational leadership showed both direct and indirect associations with sports performance through BPN satisfaction, less burnout and competitive anxiety, and an increase in life satisfaction and positive affect. To better understand the relationship between transformational leadership and motivational variables mediating well- and ill-being, among others, these authors recommend further research considering culture, age, or sex differences.

Additionally, simultaneously studying the three figures of social influence (i.e., coaches, fathers, and mothers) allows us to capture complex, multivariate interactions between contexts and figures, and to detect possible overlaps between these figures. Other variables, including BPN satisfaction and thwarting, can also benefit from these advantages.

As far as we know, the previous literature has not studied the relationships between transformational leadership, BPN satisfaction and frustration, and their implications for the development of burnout among youth athletes, taking into account, simultaneously, the contexts of social influence from sports (i.e., coaches) and family (i.e., fathers and mothers).

**Figure 1:**
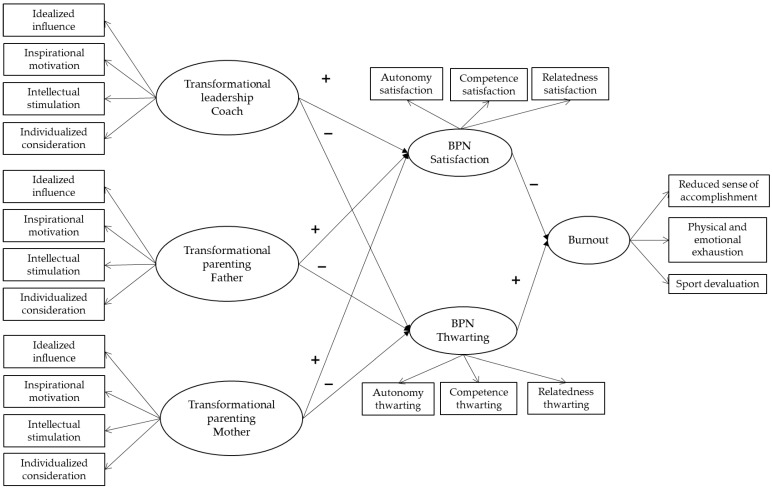
Hypothesized structural model of the associations between transformational leadership styles, basic psychological need (BPN) satisfaction, and BPN thwarting and burnout. Age and sex were used as control variables, but for the sake of clarity, they were not included in the Figure. + means positive relationship, and − means negative relationship.

Therefore, the aim of this study was to examine how the transformational leadership styles of adolescent basketball players’ key social agents, including influential figures in the sport context (i.e., coaches) and in the family context (i.e., fathers and mothers), relate to players’ burnout. The study also explores the mediating role of players’ BPN satisfaction and thwarting in these relationships. Drawing on BPNT and extending findings from previous research, this study provides a joint examination of mechanisms operating in both the sport (i.e., coaches) and family (i.e., parents or equivalent social agents) contexts and burnout during formative years. The following hypotheses will be tested (see Figure 1):

**Hypothesis 1.** 
*Coaches’ transformational leadership and fathers’ and mothers’ transformational parenting will be positively associated with players’ BPN satisfaction and negatively associated with BPN thwarting.*

**Hypothesis 2.** 
*BPN satisfaction will be negatively associated with burnout, whereas BPN thwarting will be positively associated with burnout.*

**Hypothesis 3.** 
*BPN satisfaction and BPN thwarting will mediate the relationships between coaches’ transformational leadership and players’ burnout, as well as the relationship between transformational parenting (fathers and mothers) and players’ burnout.*

## 2. Materials and Methods

### 2.1. Study Design

A non-experimental cross-sectional study was conducted in accordance with the Declaration of Helsinki and approved by the Institutional Review Board (or Ethics Committee) of the University of Valencia (protocol code 1870190—3 February 2022).

### 2.2. Participants

Participants were 540 basketball players (65.9% male) aged 11–18 years (M = 13.87, SD = 1.34). The sample comprised 62% in early adolescence (11–14 years), 31% in late adolescence (15–18 years), and 7% who did not report their age. Participants were recruited through convenience sampling from 50 teams across five basketball clubs in the province of Valencia (Spain). Each team was led by a head coach, and most players (75%) had trained under their current head coach for a single season.

### 2.3. Instruments

Coaches’ transformational leadership style was measured using the Spanish version of the Transformational Teaching Questionnaire (TTQ) [43,44], adapted for basketball. The questionnaire begins with the stem “My basketball coach…” and includes 16 items rated on a 6-point Likert scale ranging from 1 (Not at all) to 6 (Frequently). It comprises four subscales (four items each) reflecting players’ perceptions of their coach’s transformational behaviors: idealized influence (e.g., “Acts as a person that I look up to”), inspirational motivation (e.g., “Is enthusiastic about what I am capable of achieving”), intellectual stimulation (e.g., “Encourages me to look at issues from different sides”), and individualized consideration (e.g., “Shows that he/she cares about me”). The questionnaire has demonstrated adequate psychometric properties in samples of Spanish and Argentinian adolescents [14,44].

The Transformational Parenting Questionnaire (TPQ) [17] was used in its Spanish version [21] to measure players’ perceptions of transformational parenting styles. The TPQ begins with the stem “My father/mother…”, and items are answered on a 6-point Likert scale ranging from 1 (Strongly disagree) to 6 (Strongly agree), with higher scores indicating higher perceived transformational parenting. Each item was answered twice, once referring to the father and once to the mother. The instrument consists of 16 items across four subscales reflecting the dimensions of transformational parenting: idealized influence (e.g., “Behaves as someone I can trust”), inspirational motivation (e.g., “Is optimistic about what I can accomplish”), intellectual stimulation (e.g., “Gets me to think for myself”), and individualized consideration (e.g., “Helps me when I am struggling”). Previous research has demonstrated adequate validity and reliability for this [21].

The Spanish version of the Basic Psychological Need Satisfaction and Frustration Scale—Children (BPNSFS-Child) [45,46,47] was used to assess athletes’ BPN satisfaction and BPN thwarting. The instrument was adapted to the basketball context and begins with the stem “When I practice basketball…”. Responses are provided on a 6-point Likert scale ranging from 1 (Totally False) to 6 (Totally True). The scale includes 24 items divided into six subscales (four items each): autonomy satisfaction (e.g., “I feel free to choose which activities I do”), competence satisfaction (e.g., “I am good at what I do”), relatedness satisfaction (e.g., “I feel close to and connected with the people who are important to me”), autonomy thwarting (e.g., “I feel forced to do many things that I actually do not want to do”), competence thwarting (“I feel insecure about what I am able to do”), and relatedness thwarting (e.g., “I feel excluded from the group I want to be a part of”). Previous research has demonstrated the instrument’s factorial validity and reliability [45].

The Spanish version of the Athlete Burnout Questionnaire (ABQ) [36,48] was used to assess athletes’ burnout levels. The instrument begins with the stem “During the last month…”, and consist of 15 items divided into three subscales of five items each: reduced sense of accomplishment (e.g., “It seems that no matter what I do, I don’t perform as well as I should”), physical and emotional exhaustion (e.g., “I feel exhausted by the physical and mental demands of the sport”), and sport devaluation (e.g., “I have negative feelings and thoughts towards basketball”). Items are rated on a 6-point Likert scale ranging from 1 (Never) to 6 (Always). Higher scores indicate higher levels of burnout, except for items 1 and 14, which are reverse-worded for the reduced sense of accomplishment dimension (lower scores reflect higher burnout). The instrument has demonstrated adequate reliability and validity [33,49,50].

Age and sex were considered as control variables. Previous research has suggested that age and sex can modulate perceptions of transformational leadership in coaches and parents, as well as perceptions of sport burnout [14,51,52].

### 2.4. Procedure

Basketball clubs were contacted to present the aims of the study and invite participation. After institutional approval, parents or legal guardians and athletes provided informed consent. Confidentiality of all responses was guaranteed.

Data were collected at the onset of the competitive season, from October to December 2021. Adolescents voluntarily and anonymously completed the questionnaires during a 15 min period before or after training sessions. At least one researcher was present at all times to address any questions and to notify participants when they skipped a question, so that there was no missing data. Response rates were calculated based on information provided by the clubs. The final participation rate was 86% for players, 100% for coaches, and 76% for parents.

### 2.5. Statistical Analysis

The statistical analyses were conducted using G*Power version 3.1 [53], SPSS version 21.0 for Windows [54], and Mplus version 6.12 [55].

The required sample size was determined using the G*Power program. Assuming a small effect size (f2 = 0.03) for up to five predictors and a type I error probability of 0.05, a sample size of 434 individuals would be required to achieve a statistical power of 0.80 [53]. Thus, the sample size is considered to have sufficient statistical power to detect relevant relationships between the models’ variables. Descriptive analyses (mean and standard deviations), internal reliability estimates (Cronbach’s alpha), and correlation analyses (Pearson’s r) were conducted using SPSS. Regression assumptions (homoscedasticity, normality, independence of the residuals, and collinearity) were assessed and were met satisfactorily [56].

Confirmatory factor analyses (CFA) of the study instruments and structural equation modeling (SEM) to test the relationships among the study variables were conducted using Mplus. The data were analyzed using the robust maximum likelihood estimation method, as this approach allowed for reliable parameter estimates and adequate model fit even in the presence of deviations from normality. The independent variables in the hypothesized model were the coach’s transformational leadership, transformational parenting of the father, and transformational parenting of the mother. The mediating variables were BPN satisfaction and thwarting. The dependent variable was athlete burnout. We used the dimensions of each scale as indicators of latent variables and age and sex as control variables (see Figure 1). Model fit was evaluated using the following indices: chi-squared (χ^2^), comparative fit index (CFI), Tucker–Lewis index (TLI), root mean square error of approximation (RMSEA), and standardized root mean square residual (SRMR). Fit criteria followed Hu and Bentler [57], with acceptable values defined as CFI and TLI greater than 0.90, RMSEA lower than 0.08, and SRMR lower than 0.08.

Analyses were conducted in three steps. First, the hypothesized SEM model was tested. Second, to examine the mediated or indirect effects (transformational leadership—BPN satisfaction and thwarting—burnout), we employed the bias-corrected bootstrap confidence interval method, as implemented in Mplus. If the confidence interval does not include zero, the null hypothesis of no mediation is rejected, thereby providing empirical support for the indirect effect. Finally, alternative models were compared to evaluate full versus partial mediation. To compare the alternative model’s goodness of fit, the incremental fit indices were estimated. Regarding criteria for interpreting incremental fit indices, it was suggested that a difference of 0.01 or less between values of CFI (ΔCFI) and TLI (ΔTLI) reflects practically irrelevant differences between models. Similarly, it was suggested that RMSEA increases of <0.015 between alternative models indicate irrelevant differences, and, therefore, the most parsimonious model should be selected.

## 3. Results

### 3.1. Confirmatory Factor Analysis, Reliability, and Descriptive Statistics

The results of the four-factor model solution adequately fit the data for transformational leadership of the coach (CFI = 0.94, TLI = 0.92, RMSEA = 0.05, and SRMR = 0.05), the four-factor model tested for the transformational parenting of the father (CFI = 0.96, TLI = 0.96, RMSEA = 0.04, and SRMR = 0.04), the four-factor model tested for the transformational parenting of the mother (CFI = 0.95, TLI = 0.94, RMSEA = 0.04, and SRMR = 0.04), the six-factor model for the BPNSFS-Child questionnaire—including the corresponding three dimensions for BPN satisfaction and BPN thwarting scales (CFI = 0.95, TLI = 0.94, RMSEA = 0.03, and SRMR = 0.04)—and the three-factor model for the burnout scale (CFI = 0.90, TLI = 0.88, RMSEA = 0.07, and SRMR = 0.06). The alpha coefficients, which ranged from 0.81 to 0.94, indicated satisfactory reliability for all study variables (see Table 1).

Table 1 presents the descriptive statistics (mean and standard deviation), internal reliability coefficients, and correlations among the study variables. Data normality was also examined, showing skewness values ranging from −2.49 to 0.87 and kurtosis values from 0.02 to 8.53.

Overall, the players reported that both their coaches and parents frequently displayed transformational behaviors. They also indicated a high degree of satisfaction of their BPN, whereas their BPN thwarting was low. Burnout levels among the players were also low. All correlations among the variables were statistically significant (*p* < 0.01) and aligned with the expected patterns (see Table 1).

### 3.2. Structural Equation Modeling

**Figure 2:**
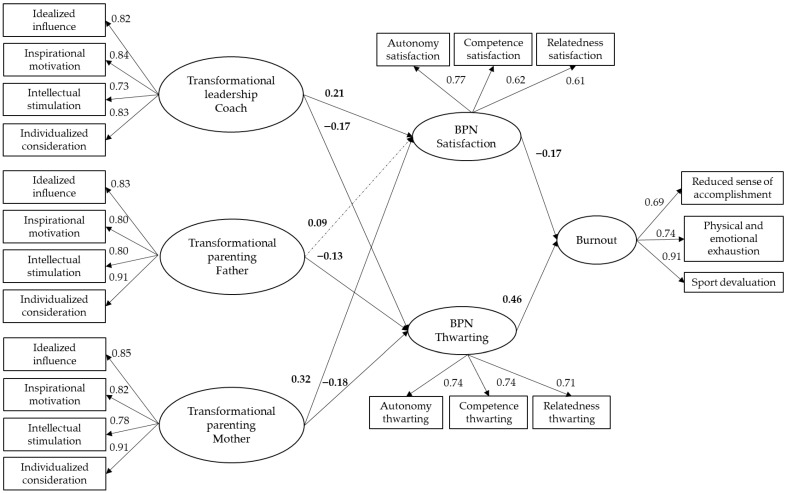
Standardized solution for the hypothesized model of the relationships between transformational leadership styles, basic psychological needs (BPN) satisfaction, and BPN thwarting and burnout. Factor indicators are represented, and values are shown. The correlation (r) value between the error of BPN satisfaction and BPN thwarting is −0.58, *p* < 0.01. All values are significant at *p* < 0.05; the dashed line indicates a non-significant relationship.

The findings indicated that coaches’ transformational leadership was positively associated with players’ satisfaction of their BPN and negatively related with BPN thwarting. Fathers’ transformational parenting was negatively associated with BPN thwarting, although no significant relationship emerged with BPN satisfaction. In contrast, mothers’ transformational parenting was positively associated with BPN satisfaction and negatively associated with BPN thwarting. Finally, BPN satisfaction was negatively associated with burnout, whereas BPN thwarting was positively associated with burnout (see Figure 2). The model accounted for 24% of the variance in BPN satisfaction, 14% of the variance in BPN thwarting, and 35% of the variance in burnout, and it exhibited adequate fit indices: χ ^2^ (207) = 551.96, *p* < 0.001; CFI = 0.944; TLI = 0.931; RMSEA = 0.058; and SRMR = 0.053. The χ^2^ value was statistically significant, indicating a non-satisfactory fit of the proposed model. However, this result is common in large samples, such as the one used in the present study, and was therefore not considered problematic.

None of the control variables considered showed a significant relationship with burnout (age: β = 0.04, *p* = 0.34; sex: β = −0.03, *p* = 0.51), indicating that the perception of burnout is the same for both males and females, and for those in different age groups. However, age and sex were negatively associated with coaches’ transformational leadership (β = −0.14, *p* = 0.01 and β = −0.12, *p* = 0.02, respectively), and with fathers’ transformational parenting (β = −0.10, *p* = 0.04 and β = −0.14, *p* = 0.01, respectively), indicating that males and younger children perceive a greater level of transformational leadership from their coach and from their father. Finally, control variables were not associated with mothers’ transformational parenting (age: β = −0.11, *p* = 0.06; sex: β = −0.05, *p* = 0.26), which means that children perceive the same transformational parenting from their mother, regardless of whether they are male or female, or regardless of their age.

### 3.3. Indirect Effects Model (Mediation Analysis)

Table 2 presents the association of BPN satisfaction and BPN thwarting with athletes’ burnout. BPN thwarting emerged as a significant mediator in the relationship between coaches’ transformational leadership style and players’ burnout. Both BPN satisfaction and BPN thwarting emerged as significant mediators in the relationships between mothers’ transformational parenting and children’s burnout. In contrast, for the relationship between the fathers’ transformational parenting and children’s burnout, none of the mediators emerged as significant.

Furthermore, a comparison was made between the indirect effects of the coaches’ transformational leadership and the mothers’ transformational parenting on burnout via BPN thwarting to ascertain whether their magnitudes were different. We used the model test command in Mplus and the corresponding Wald test. This test was not statistically significant (W(1) = −0.05, *p* = 0.32), indicating that the two indirect effects were not statistically different from each other, and the mediator contributed equally to explaining the relationship between coaches’ and mothers’ transformational leadership and burnout. We also compared the two significant indirect effects from transformational leadership of coaches to burnout via BPN thwarting and transformational parenting of mothers to burnout via BPN satisfaction. The Wald test was statistically significant (W(1) = 0.12, *p* = 0.01), indicating that the first indirect effect (through BPN thwarting) was stronger than the other (through BPN satisfaction). Finally, a comparison was made between the two significant indirect effects from transformational leadership of mothers to burnout via BPN satisfaction and via BPN thwarting. The Wald test was statistically significant (W(1) = −0.17, *p* = 0.01), indicating that the second indirect effect (through BPN thwarting) was stronger than the other (through BPN satisfaction).

### 3.4. Direct Effects Model

To evaluate the direct effects of the three influential figures’ leadership styles on burnout, and to determine whether the mediation in the previous model was full or partial, one alternative model was subsequently tested, adding the three direct paths.

The partially mediated model showed a satisfactory fit to data: χ^2^ (204) = 461.39, *p* < 0.001; CFI = 0.943; TLI = 0.930; RMSEA = 0.051; and SRMR = 0.050. The CFI, TLI, and RMSEA values obtained for the fully mediated model showed no relevant difference when compared with those obtained for the partially mediated model (ΔCFI = 0.001; ΔTLI = 0.001; ΔRMSEA = 0.007).

The coaches’ transformational leadership style showed a significant negative direct effect (β = −0.11, *p* = 0.04) on the athletes’ burnout. Combined with the mediating role of BPN thwarting, this finding indicated partial mediation between the variables. The fathers’ transformational parenting did not have a significant direct effect (β = 0.02, *p* = 0.82) on their children’s burnout. Finally, the mothers’ transformational parenting did not have a significant direct effect (β = −0.02, *p* = 0.87) on their children’s burnout. Nonetheless, BPN satisfaction and BPN thwarting demonstrated a significant indirect effect, acting as mediators and indicating full mediation.

## 4. Discussion

The present study aimed to examine the simultaneous relationship between transformational leadership styles (i.e., transformational coaching and transformational parenting) and their associations with BPNs’ satisfaction, thwarting, and burnout. To this end, the relationships of the three primary agents of athletic socialization (i.e., coaches, fathers, and mothers) were studied in a sample of Spanish adolescent basketball players. Additionally, the mediational role of BPN satisfaction and thwarting between transformational styles and adolescents’ burnout was addressed. Drawing on BPNT and extending findings from previous research, this study provides a joint examination of mechanisms operating in both the sport (i.e., coaches) and family (i.e., parents or equivalent social agents) contexts, and burnout during formative years.

The results indicated a positive association between coaches’ transformational leadership and players’ BPN satisfaction, and a negative association with BPN thwarting. These findings are consistent with previous research reporting similar relationships between transformational leadership and BPN satisfaction [58,59]. They are also aligned with early conceptualizations of transformational leadership, in which Burns [60] defined a transformational leader as someone who “seeks to satisfy higher needs and engages the full potential of the follower” (p. 4). In a similar vein, Bass [61] emphasized that fulfilling followers’ emotional needs is a key characteristic of transformational leadership. In the sport domain, previous studies [12,30,62] have suggested that transformational leaders foster athletes’ BPN satisfaction by shaping their psychosocial development.

On the other hand, the expected positive association between fathers’ transformational parenting and athletes’ BPN satisfaction was not significant. However, a significant negative association was found between fathers’ transformational parenting and athletes’ BPN thwarting. In contrast, a positive association was observed between mothers’ transformational parenting and athletes’ BPN satisfaction, and a negative association with BPN thwarting. These results may be understood in light of prior research indicating that children perceive differences in autonomy support from fathers and mothers. In general, children tend to perceive that mothers foster their autonomy to a greater extent than fathers do [21,63]. Thus, when both parental figures are considered, fathers’ associations with their children’s BPN satisfaction appear to be less pronounced than those of mothers. These BPN findings do not support Hypothesis 1.

The results of this study show negative associations between players’ BPN satisfaction and burnout and positive associations between BPN thwarting and burnout. These results support Hypothesis 2 and align with the tenets of BPNT [24], which posit that the satisfaction of BPN fosters well-being (e.g., subjective vitality) [64], while BPN thwarting contributes to ill-being (e.g., burnout) [65]. Previous literature offered empirical support in the sports domain [66,67]. For example, in a sample of adolescent football players, González and colleagues [66,67] found that those players who experienced higher levels of competence, autonomy, and relatedness reported higher subjective vitality and self-esteem, alongside lower burnout, whereas those who experienced BPN thwarting showed the opposite pattern.

Additionally, we tested the mediating role of BPN satisfaction and thwarting between a transformational coaching style and a transformational parenting style and burnout. Our results show, for coaches, direct and indirect associations. That is, coaches’ leadership style shows a direct and negative association with players’ burnout, as well as through BPN thwarting, confirming a partial mediation. These results offer empirical evidence of direct associations that previous studies suggested but did not find [12,13]. Thus, Stenling and Tafvelin [12] found a full mediation of BPN satisfaction between transformational coaching and well-being in adolescent floorball players. In the same vein, Yildirim and colleagues [13] found a full mediation of BPN satisfaction between transformational coaches and players’ burnout in a sample of adolescent football players. Therefore, our results extend findings from the literature to better understand the role of transformational coaching and burnout, as indicator of ill-being.

Regarding parents’ social influence, our data confirmed the mediational role of BPN satisfaction and thwarting between mothers’ transformational parenting and burnout. In our sample, this association is fully mediated by BPN satisfaction and thwarting. That is, the more transformational the mother’s parenting style, the more satisfied her children’s BPN, and her children have less burnout. On the other hand, transformational mothers tend to have a lower probability of thwarting their children’s BPN, and low levels of BPN frustration correlate with their children having fewer opportunities to suffer burnout.

Our data suggests that BPN satisfaction and thwarting are the main mechanisms to understand the development of adolescents’ burnout. That is, in the case of mothers, adopting a transformational style reduces the possibility of having adolescent children with burnout, but this association is due to the role of the satisfaction and frustration of their children’s BPN. Moreover, and reinforcing the aforementioned idea, additional analysis comparing mediational associations in the model (i.e., BPN satisfaction and thwarting mediating mothers’ transformational parenting and burnout, and BPN thwarting mediating transformational coaching and burnout), suggests that the most powerful association with adolescent burnout is the mediation of BPN thwarting in the case of mothers’ transformational parenting. Although our data suggests that transformational fathers are less likely to thwart the BPN of their children, the relationship between fathers’ transformational parenting and their children’s burnout is not confirmed in our sample. Therefore, Hypothesis 3 is not confirmed.

Overall, our results are aligned with the previous literature that suggested that, when simultaneously studying both parenting roles (father and mother, or equivalent roles) in a Mediterranean cultural context, mothers have a predominant role in relation to their children’s burnout [14,21]. Thus, Alvarez and colleagues [21] found that transformational parenting was associated with lower levels of burnout among adolescent football players, particularly through mothers’ autonomy support. Celsi and colleagues [14] reported similar maternal–paternal distinctions in youth athletes’ positive development. Previous literature has reported a tendency for mothers to adopt a more facilitating role [68], reinforced by the unconditional support perceived by their children [69], which could be a possible explanation for the different roles of mothers and fathers in the families studied. Moreover, the mother–child bond provides an “umbrella effect” that encompasses essential behaviors (e.g., emotional, logistical, and financial support), which contribute to a child’s optimal development [69,70].

Simultaneously considering coach leadership and fathers’ and mothers’ transformational parenting’s direct associations with players’ burnout, the current study only found direct associations between coaches’ leadership and players’ burnout. This direct association of leadership should be interpreted in regard to the central and critical role of coaches in the sports setting [71]. These results do not imply that the figures studied are more or less important in relation to player burnout. In fact, what our data indicate is that when we include all three figures simultaneously, the direct association between leadership and burnout remains with the figure that the player has closest to their sporting practice, which is the coach.

### 4.1. Limitations and Future Research

The present study has limitations that should be addressed in future research. First, the cross-sectional design prevents causal inferences and precludes interpreting mediation paths as developmental mechanisms; longitudinal studies are needed to establish temporal precedence and examine the stability of these associations over time. Second, the sample consisted exclusively of Spanish adolescent basketball players, which implies limitations affecting the generalizability of our results; therefore, future studies could include athletes from different cultures, sports, and age groups (e.g., childhood, adolescence, and adulthood). Third, the gender of both coaches and athletes was not examined in the present study, yet it may influence the observed relationships and should be examined in future research. Nevertheless, in this study, the age and sex of the players were considered as control variables. Fourth, most of the reviewed literature on this subject studies team athletes. It might be advisable to study these variables in individual sports. Fifth, data were collected via self-reporting, which may introduce “common method bias” [72]; it would be valuable for future research to incorporate objective measures, such as researcher observations of coaches’ leadership behaviors. Finally, as only quantitative data were collected, future research could benefit from qualitative methods, such as focus groups with athletes and coaches, to complement and contextualize the quantitative results.

### 4.2. Practical Implications

With the necessary caution, due to the cross-sectional and correlational nature of the study, but considering that our work provides evidence in line with the previous literature, we encourage sporting club administrators to assess the leadership styles of their current coaches. In the absence of transformational behaviors, the implementation of specific training programs could be considered to promote these behaviors. Alternatively, the establishment of a transformational leadership profile would be considered as a criterion for selecting future coaches.

The awareness of the parenting styles employed by athletes’ parents may enable sports clubs to develop family-oriented interventions. These interventions must be empirically validated and aim to promote transformational parenting behaviors.

## 5. Conclusions

This study expands knowledge about the mechanisms that operate in the relationships between figures of social influence in sport from two fundamental contexts in the development of young athletes: sports (i.e., coaches) and family (i.e., parents). Specifically, we have found two figures to be important in relation to players’ burnout: coaches and mothers. Thus, the transformational leadership style of coaches is shown to play a relevant role in relation to the sports burnout of adolescent basketball players, both directly and through the frustration of players’ BPN.

In line with previous studies, the figure of the mother is shown to be more closely related to the burnout of their children when we evaluate the paternal and maternal figures simultaneously.

Finally, the positive role of transformational styles (transformational coaching and transformational parenting) in relation to the satisfaction of BPN and burnout in adolescent athletes was confirmed.

## Figures and Tables

**Table 1:** Descriptive statistics, reliability, and bivariate correlations of the study variables (*n* = 540).

Variables	Mean	SD	Alpha	1	2	3	4	5
1. Transformational leadership coach	4.99	0.69	0.91	-				
2. Transformational parenting father	5.32	0.75	0.94	0.23 **	-			
3. Transformational parenting mother	5.45	0.64	0.92	0.29 **	0.65 **	-		
4. BPN Satisfaction	5.00	0.60	0.81	0.27 **	0.29 **	0.37 **	-	
5. BPN Thwarting	2.76	0.94	0.87	−0.21 **	−0.26 **	−0.27 **	−0.48 **	-
6. Burnout	2.21	0.83	0.89	−0.26 **	−0.19 **	−0.24 **	−0.38 **	0.47 **

Note. Range variables = 1–6; ** *p* < 0.01.

**Table 2:** Path coefficients and indirect effects for mediation model with burnout as outcome (*n* = 540).

Predictors	Mediators	Estimation	Lower Limit	Upper Limit
Transformational leadership coach	BPN Satisfaction	−0.03	−0.10	0.00
	BPN Thwarting	0.06	0.02	0.12
Transformational parenting father	BPN Satisfaction	−0.01	−0.07	0.01
	BPN Thwarting	0.03	−0.02	0.09
Transformational parenting mother	BPN Satisfaction	−0.06	−0.17	−0.01
	BPN Thwarting	0.11	0.04	0.22

Note. BPN = Basic psychological needs.

## Data Availability

All data used in this study are presented in the manuscript.
